# Delayed manifestation of severe coronary artery injury/stenosis associated with cavo-tricuspid isthmus ablation: a case report

**DOI:** 10.1093/ehjcr/ytae701

**Published:** 2024-12-30

**Authors:** Honsa Kang, Masao Takemoto, Takanori Watanabe, Kiyoshi Hironaga

**Affiliations:** Cardiovascular Centre, Fukuoka City Hospital, 13-1 Yoshizukahon-machi, Hakata-ku, Fukuoka 812-0046, Japan; Cardiovascular Centre, Social Medical Corporation Steel Memorial Yawata Hospital, 1-1-1 Haruno-machi, Yahata-higashi-ku, Kitakyushu 805-8508, Japan; Cardiovascular Centre, Fukuoka City Hospital, 13-1 Yoshizukahon-machi, Hakata-ku, Fukuoka 812-0046, Japan; Cardiovascular Centre, Fukuoka City Hospital, 13-1 Yoshizukahon-machi, Hakata-ku, Fukuoka 812-0046, Japan

**Keywords:** Ablation, Angina, Atrial flutter, Cavo-tricuspid isthmus, Coronary injury, Delayed manifestation, Case report

## Abstract

**Background:**

Radiofrequency ablation (RFA) procedures including cavo-tricuspid isthmus (CTI) ablation have proven to be safe and effective therapies for the treatment of many cardiac tachyarrhythmias. The incidence of coronary arterial injury (CAI) associated with RFA including CTI ablation is estimated to occur in <0.1% of patients. Most instances of CAI occur during ablation procedures or within a few weeks after RFA.

**Case summary:**

We report a case of delayed manifestation of CAI of the right coronary artery 1 year after RFA, likely associated with a CTI ablation. The coronary angiography and intravascular ultrasound images revealed significant stenotic lesions primarily consisted of heterogeneous fibrous plaques including few echolucent lesions that consisted of a relatively smaller lipid or necrotic core without echo-attenuated plaques consisting of a fibroatheroma with a necrotic core or pathological intimal thickening with a lipid pool, and corresponded to the site of the CTI ablation. The patient remained stable without any symptoms 6 months post-percutaneous coronary intervention at that site.

**Discussion:**

Physicians should consider the possibility of CAI associated with RFA procedures involving ablation near the coronary arteries (CAs) in patients presenting with chest discomfort after RFA, even when the presentation is remote from the index procedure. Unanticipated anatomic variations can predispose to CAIs. Therefore, awareness of the relationship between CA course and anatomical ablation site before RFA may be important to prevent CAIs and improve procedural safety.

**Learning points**

- Physicians should consider the possibility of coronary arterial injury (CAI) associated with radiofrequency ablation (RFA) procedures involving ablation near the coronary arteries (CAs) in patients presenting with chest discomfort after RFA, even when the presentation is remote from the index procedure.
- Although CAI is a rare complication, operators should have general awareness of the relation between the course of the CAs and anatomical sites of ablation.

## Introduction

Radiofrequency ablation (RFA) procedures including cavo-tricuspid isthmus (CTI) ablation have been proven safe and effective for the treatment of many cardiac tachyarrhythmias.^1^ The incidence of coronary arterial injury (CAI) associated with RFA including CTI ablation^1^ is estimated to occur in <0.1% of patients.^2–4^ Most instances of CAI occur during ablation or within a few weeks after RFA.^2–4^ Herein, we report a case of delayed manifestation of CAI of the right coronary artery (RCA) 1 year after RFA, which was likely associated with a CTI ablation.

## Summary figure

**Figure:**
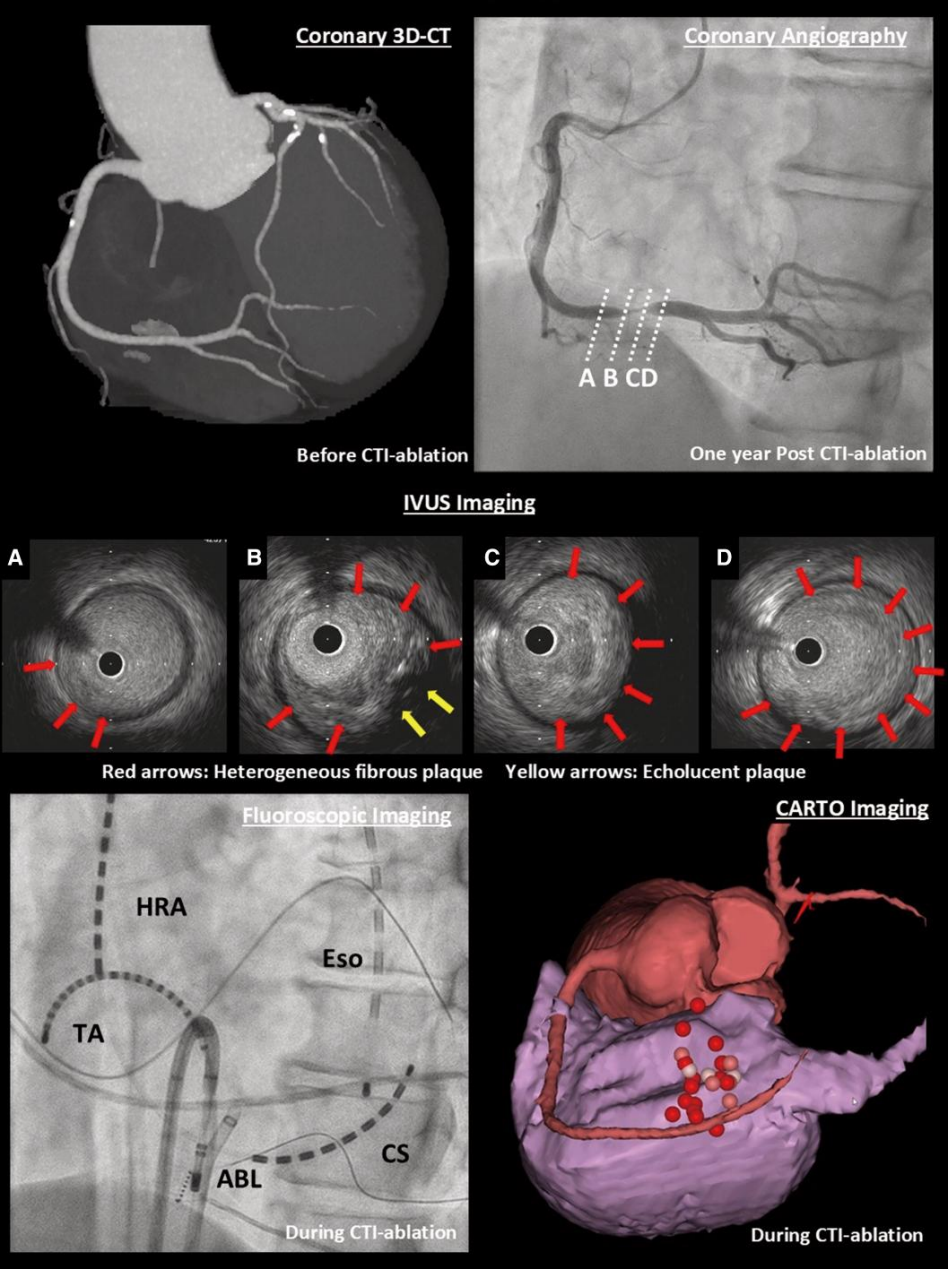


## Case presentation

**Figure 1:**
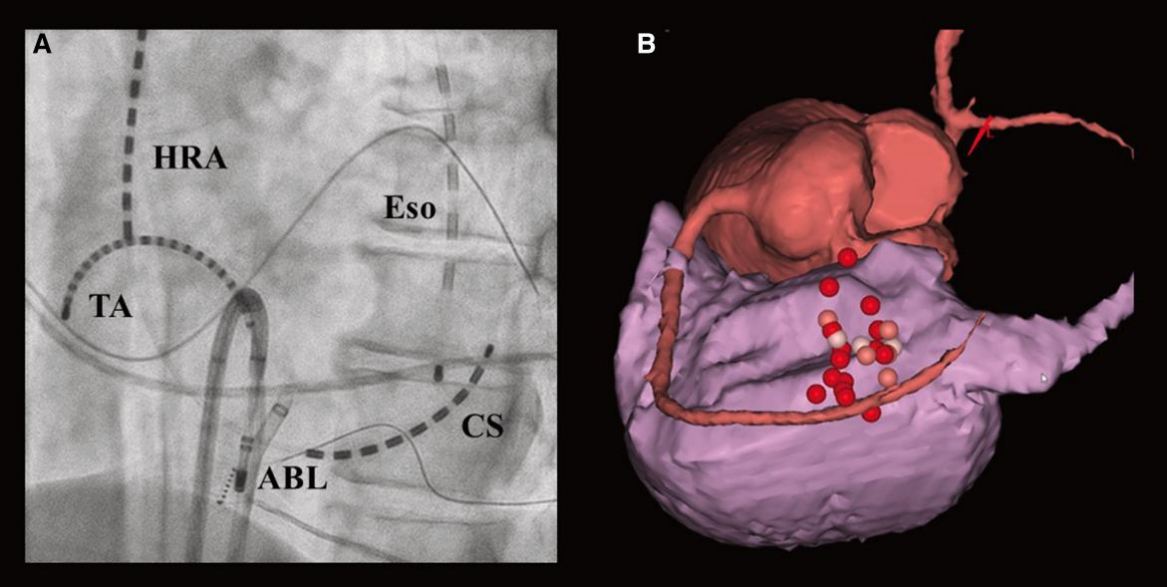
The fluoroscopic image (*A*) demonstrates the ablation catheter placed on the cavo-tricuspid isthmus. CS, coronary sinus; Eso, oesophageal temperature probe; HRA, high right atrium; TA, tricuspid annulus. Left anterior oblique caudal view of the 3D anatomical mapping (CARTO3^TM^, Johnson&Johnson) (*B*). The tags indicate the points where the radiofrequency energy was deployed.

**Figure 2:**
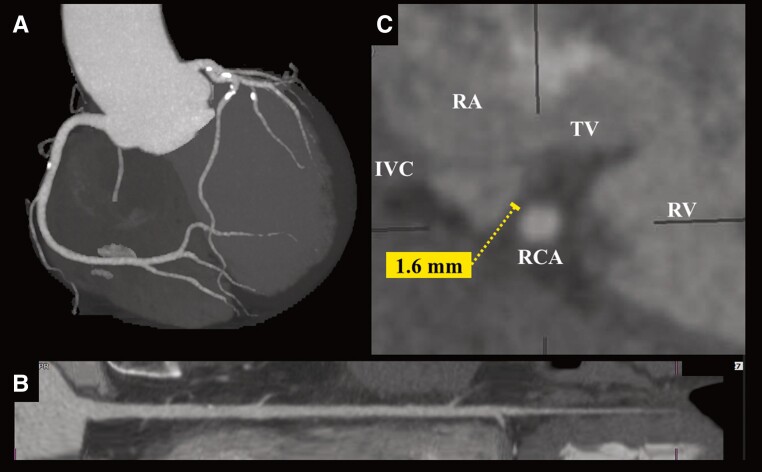
3D coronary computed tomography imaging shown in the left anterior oblique view (*A*), straight view of the right coronary artery (*B*), and trans-axis cross-sectional view at the level of the cavo-tricuspid isthmus (CTI) ablation line (*C*) before the cavo-tricuspid isthmus ablation. IVC, inferior vena cava; RA, right atrium; RCA, right coronary artery; RV, right ventricle; TV, tricuspid valve.

**Figure 3:**
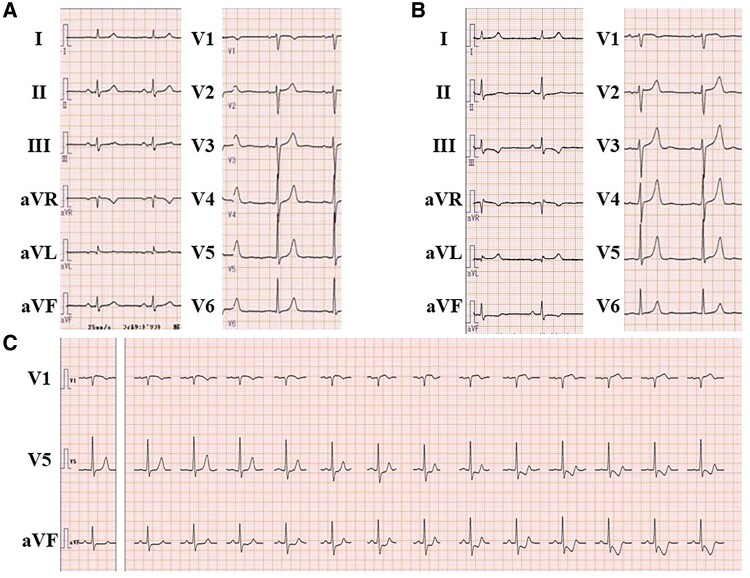
The 12-lead electrocardiograms after ablation 1 year previously (*A*) and on admission (*B*). The electrocardiogram during exercise stress testing (*C*).

**Figure 4:**
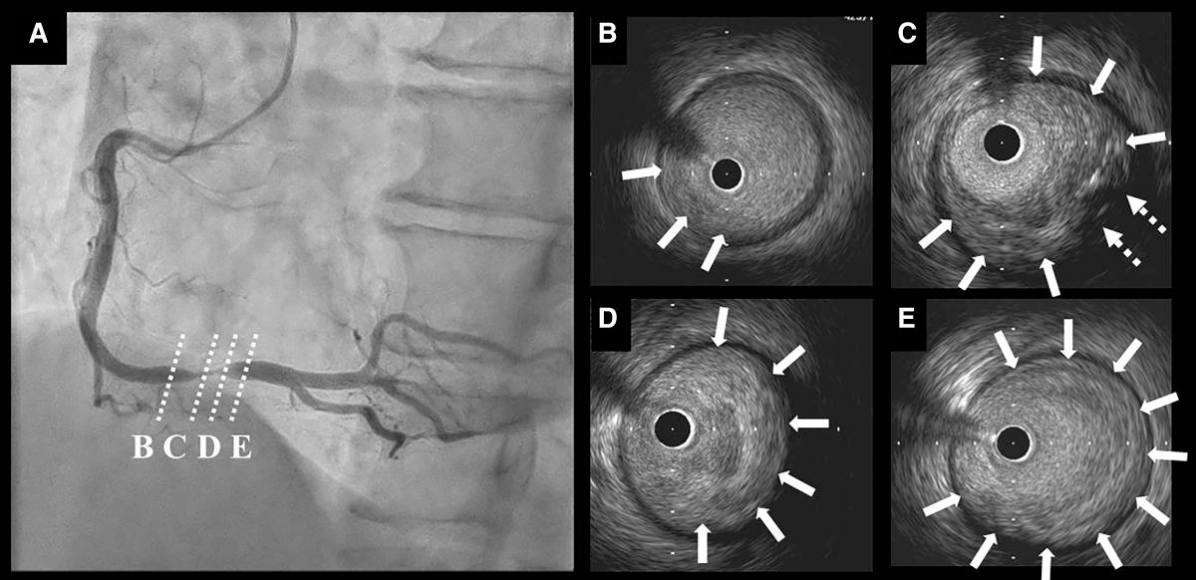
The coronary angiogram of the right coronary artery in the left anterior oblique view (*B*). The intravascular ultrasound (IVUS) images (*B–E*) reveal a significant stenosis (*C, D*), which corresponds to the site of the cavo-tricuspid isthmus ablation. Intravascular ultrasound also demonstrated that this stenotic lesion primarily consisted of heterogeneous fibrous plaque (solid arrows) including few echolucent lesions (dashed arrows).

A 64-year-old man presented with a chief complaint of exertional chest pain and was admitted to our hospital to undergo coronary angiography. He had hypertension and was an ex-smoker but had no other risk factors for coronary artery (CA) disease, such as diabetes mellitus, rheumatic diseases, or any other significant medical history, except for a history of colonoscopic polypectomy, and no family history of cardiovascular disease (CVD). He received medications included rivaroxaban 15 mg, amlodipine 2.5 mg once daily, and bisoprolol 2.5 mg twice daily. One year earlier, he had undergone a CTI ablation for CTI-dependent atrial flutter (*Figure 1A* and *B*). Then, levels of low- and high-density lipoprotein cholesterol were 104 mg/dL (normal range: 70–139 mg/dL) and 67 mg/dL (normal range: 41–96 mg/dL), respectively. A coronary computed tomography angiogram before the RFA had shown no significant CA stenosis (*Figure 2A* and *B*). Retrospectively, the minimum distance between the RCA and CTI ablation line was 1.6 mm (*Figure 2C*). Radiofrequency (RF) energy was applied with a ThermoCool SmartTouch^TM^ Surround Flow catheter (Johnson&Johnson) with impedance of 90–110 Ω, maximum temperature set to 43°C, and a maximum power of 35 W, in the paraseptal region of the CTI (*Figure 1A* and *B*). The RF application was delivered for 20–30 s per point. A 12-lead electrocardiogram after RFA revealed sinus rhythm with no ST-T changes (*Figure 3A*). The patient has experienced no symptoms or arrhythmias for 1 year. However, a few months before readmission, he began experiencing episodes of chest pain on exertion. A 12-lead electrocardiogram revealed sinus rhythm with new T inversions in leads III and aVF (*Figure 3B*). Echocardiography revealed a normal left ventricular ejection fraction of 65%. His exercise stress testing showed a downward sloping ST-segment depression exceeding 0.2 mV in leads II, III, aVF, V5, and V6 (*Figure 3C*) accompanied by chest pain, indicative of effort angina pectoris. Thus, intensification of medical therapies including the addition of aspirin 100 mg and rosuvastatin 5 mg were started 10 days prior to admission. Upon admission, his blood pressure was 115/80 mmHg and heart rate was regular at 63 beats/min. Precordial auscultation revealed normal cardiac and respiratory sounds. His body mass index was 22.0 kg/m^2^, and his levels of B-type natriuretic peptide, serum creatinine, low- and high-density lipoprotein cholesterol, and fasting blood glucose levels were 61.2 pg/mL (normal range: <18.4 pg/mL), 0.93 mg/dL (normal range: 0.65–1.09 pg/mL), 119 mg/dL (normal range: 70–139 mg/dL), 66 mg/dL (normal range: 41–96 mg/dL), and 93 mg/dL (normal range: 69–104 mg/dL), respectively. His estimated CVD risk score was moderate.^5^ Coronary angiography revealed 90% severe stenosis at the distal site of the RCA (*Figure 4A*), whereas the left CA appeared normal. Intravascular ultrasound (IVUS) images (*Figure 4B–E*) revealed significant stenosis with a minimal luminal area of 1.24 mm^2^ (*Figure 4C*), corresponding to the site of the CTI ablation (*Figures 1A* and *B* and *4A*). Intravascular ultrasound also demonstrated that the stenotic lesion primarily consisted of a heterogeneous fibrous plaque (solid arrows in *Figure 4B–E*), including few echolucent lesions (dashed arrows in *Figure 4C*) indicate healed plaque.^6^ There were no echo-attenuated plaques (*Figure 4B–E*). Subsequently, a 4.0 mm × 28 mm Xience Skypoint^TM^ stent (Abbott) was deployed. The patient remained stable without any symptoms for 6 months post-percutaneous coronary intervention and without any arrhythmia recurrence 18-month post-ablation.

## Discussion

The incidence of CAIs associated with RFA is low.^2–4^ However, when CAIs associated with RFA occurs, percutaneous coronary intervention is required in a third of patients. There is an associated risk of mortality of 5%.^2^ The RCA is the site of the CAIs in over two-thirds of all reported cases.^3^ Among these, CTI ablation^1^ accounted for the most reported CAI cases. Several studies have demonstrated the variability in the manifestation of CAI after ablation.^2–4^ Most CAIs associated with RFA of the RCA have been reported to occur during RFA procedures.^2–4^ Most of these acute responses have been attributed to coronary spasm-related coronary occlusion, dissection, haematoma, thrombosis, air embolism, or plaque rupture, as several reports have demonstrated chest pain with ST elevation after ablation.^2–4^ CAIs of the left circumflex artery were detected intra-procedurally in approximately half of the cases, whereas the other half manifested within a few weeks after RFA.^2–4^ However, there have been no reports of a delayed manifestation of CAI occurring 1 year after RFA, as observed in this case. A previous study examined the anatomic relationships between the CAs and the tricuspid or mitral annulus. The RCA was <5 mm from the CTI in 8% of patients, and the left circumflex artery was 2 mm away from the lateral mitral annulus in 24% of patients.^7^ In this case, the minimum distance between the RCA and CTI ablation line was 1.6 mm (*Figure 2C*). Recent reports^3,8^ have proposed that the risk of CAIs increased significantly for distances less than 2 mm, suggesting a high risk of CAIs in this case. Thus, because unanticipated anatomic variations can predispose to CAIs,^8^ awareness of the relation between CA course and anatomical ablation site before RFA may be important to prevent CAIs and improve procedural safety. Although the mechanism(s) underlying the delayed manifestation of CAI after RFA have not been completely elucidated, several potential mechanism(s) can be proposed. (i) As mentioned earlier, some acute CAIs have been attributed to pathophysiological vascular injuries. In this present case, however, acute CAIs might not have caused symptomatic significant coronary stenosis. (ii) Given that the minimum distance between the RCA and CTI ablation line was 1.6 mm (*Figure 2C*) in this case, RF energy may have caused the functional and morphological CAI, including of the CA media and endothelium, and vascular inflammation of the RCA. Endothelial damage and vascular inflammation can progress to atherosclerosis^9^ and develop into significant severe coronary stenosis. (iii) Another mechanism of RFA-induced CAIs is proposed to be heat-induced collagen shrinkage and subsequent vessel narrowing.^2^ The extent of CAI correlates with the amount of heat-induced denaturation of collagen fibres in the vessel wall.^2^ Replacing the CA media with a proliferating extracellular matrix can lead to severe hyperplasia and intravascular thrombosis.^2^ Generally, within several weeks after RFA, fibrosis has almost replaced the lesion.^2^ (iv) Because the patient’s estimated risk score of the CVD was moderate,^5^ these conditions may cause the acceleration of coronary stenosis. Finally, in this case, those pathophysiological changes might quietly and slowly progress a healing process without clinical symptoms resulting in a significant stenosis with healed plaque (*Figure 4B–E*) 1 year after RFA. The IVUS images demonstrated that the stenotic lesions in this case primarily consisted of heterogeneous fibrous plaques (solid arrows in *Figure 4B–E*) including few echolucent lesions that consisted of a relatively smaller lipid or necrotic core (dashed arrows in *Figure 4C*) without echo-attenuated plaques consisting of a fibroatheroma with a necrotic core or pathological intimal thickening with a lipid pool^10^ (*Figure 4B–E*). These findings may indicate that the stenotic lesions consisted of compact fibrotic changes rather than lipid-storing, supporting our hypothesis. Future studies may determine the mechanism(s) of the delayed manifestation of CAI after RFA using another imaging modality, such as optical coherence tomography and/or a pathological assessment using directional coronary atherectomy.

## Conclusions

This case report demonstrates the delayed manifestation of a symptomatic CAI 1 year after RFA. Physicians should consider the possibility of CAI associated with RFA procedures involving ablation near the CAs in patients presenting with chest discomfort after RFA, even when the presentation is remote from the index procedure.

## Data Availability

The deidentified participant data will not be shared.

## Lead author biography

Honsa Kang was born in Fukuoka. He received MD degree from University of Yamaguchi of Medicine, Yamaguchi, Japan. He belongs to the Department of Cardiovascular Medicine of Kyushu University. From April 2020 to March 2023, he worked at Miyazaki Medical Association Hospital. Since April 2023, He has been working in the Cardiology Department of Fukuoka City Hospital, primarily responsible for arrhythmia treatment.

## Notes

**Consent:** The authors confirm that written consent for the submission and publication of this case report, including the image(s) and associated text, has been obtained from the patient in line with COPE guidance.

**Funding:** None declared.
